# Single-point position and transition defects in continuous time quantum walks

**DOI:** 10.1038/srep13585

**Published:** 2015-09-01

**Authors:** Z. J. Li, J. B. Wang

**Affiliations:** 1 Institute of Theoretical Physics, Shanxi University, Taiyuan, 030006, China; 2 School of Physics, The University of Western Australia, WA 6009, Australia

## Abstract

We present a detailed analysis of continuous time quantum walks (CTQW) with both position and transition defects defined at a single point in the line. Analytical solutions of both traveling waves and bound states are obtained, which provide valuable insight into the dynamics of CTQW. The number of bound states is found to be critically dependent on the defect parameters, and the localized probability peaks can be readily obtained by projecting the state vector of CTQW on to these bound states. The interference between two bound states are also observed in the case of a transition defect. The spreading of CTQW probability over the line can be finely tuned by varying the position and transition defect parameters, offering the possibility of precision quantum control of the system.

Compared to the classical random walk, which is a memoryless Markov process, a quantum walk is unitary and time-reversible^1^,^2^. It exhibits markedly different behavior due to superposition, interference, and quantum correlations. For instance, a quantum walk can propagate quadratically faster than its classical counterpart and result in a probability distribution vastly different from the classically expected Gaussian distribution^3^. Quantum walks have become useful tools for modeling and analyzing the behavior of quantum systems, for simulating biological processes such as energy transfer in photosynthesis^4^, for studying quantum phenomena such as perfect state transfer^5^, Anderson localization^6^ and topological phases^7^, as well as for developing novel quantum algorithms in quantum information processing^8^,^9^. Experimentally, quantum walks have been implemented in a variety of systems, such as nuclear magnetic resonance^10^, trapped ions and trapped cold neutral atoms^11^,^12^, single photons in bulk^13^, fiber optics^14^, and coupled waveguide arrays^15^.

With the physical implementation of quantum walks comes the issue of disorder and decoherence. The effects of decoherence and disorder on the quantum walks have been extensively studied, for example, their transition to classical random walks under the influence of decoherence^16^,^17^,^18^. Static and dynamic disorder also alters quantum walks from ballistic spread to localization through a disruption of the interference pattern^19^,^20^,^21^,^22^,^23^,^24^. Recently, Wójcik *et al.*^25^, Li *et al.*^26^ and Zhang *et al.*^27^ investigated the localization property of one-dimensional discrete time quantum walks (DTQW) with a single-point phase defect. Motes *et al.*^28^ use a bit-flip coin at a boundary to introduce the position defects and find the walker escapes dramatically faster through the boundary. For continuous time quantum walk with defects, although a precursor work by Koster and Slater^29^ has explored quantitatively the limiting case of a single diagonal defect in a one-dimensional molecular crystal using a nearest-neighbor tight-binding model, an analytic derivation is absent to provide insight for the prevalence results relying on numerical methods. Li and Izaac^26^,^30^ have compared similar behaviors between CTQWs and DTQWs with single- and double-point defects. In this paper, we extend these works to include not only position defects but also transition defects in continuous time quantum walks, presenting analytical solutions of both traveling waves or bound states of CTQWs in position space. Here, the bound state means that the quantum walk is localized in one region of the position space with zero probability in the limit of asymptotic infinity. We use its analytical expression to discuss the associated eigenstate localization.

## Results

### The single-point defect model of CTQW

The continuous time quantum walk was first posited by Farhi and Gutmann^2^, as a quantization of the corresponding classical continuous time random walk. In CTQWs, classical probabilities are replaced by quantum probability amplitudes, with the system evolving as per the Schrödinger equation in discrete space, rather than the Markovian master equation^31^. To illustrate, we consider a classical continuous time random walk on the discrete graph *G*(*V*, *E*) described by two sets *V* and *E*. The set *V* is composed of the unordered nodes *j* and the set *E* includes the edges *e*_*jk*_ = (*j*, *k*) connecting the node *j* to the node *k*. The transition rate matrix *H* is defined as


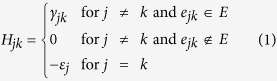


where *γ*_*jk*_ is the probability per unit time for making a transition from node *k* to node *j*. For the probability to be conservative, the constraint


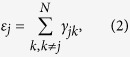


is required, where *N* is the total number of nodes in the graph. If the transition rates between any two connected nodes are the same, i.e. *γ*_*jk*_ = *γ*, the diagonal element *ε*_*j*_ = *d*_*j*_*γ* with *d*_*j*_ denoting the degree of the node *j* or the number of sites connected to node *j*. The state of the random walker is fully described by the probability distribution vector **P**(*t*), with its time evolution governed by the master equation


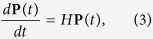


which has the formal solution **P**(*t*) = *e*^*Ht*^**P**(0).

Extending the above description to the quantum realm involves replacing the real valued probability distribution vector **P**(*t*) with a complex valued wave function 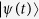 and adding the complex notation −*i* to the evolution exponent, namely


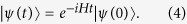


The quantum transition matrix *H*, often referred to as the system Hamiltonian, is required to be Hermitian instead of being constrained by Eq. (2). Consequently, the above time evolution is unitary, guaranteeing that the norm of 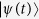 is conserved under a CTQW. Let **j** be the position operator with eigenvector 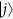. The system state vector can be expanded in the position Hilbert space with basis 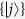, 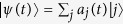 where 
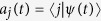
 represents the probability amplitude of the walker being found at node *j* at time *t*. The resulting probability distribution is given by 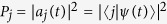.

For a CTQW on a uniform infinite line, its Hamiltonian can be expressed as


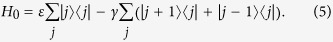


Here, each node is connected to its neighboring nodes by a constant transition rate *γ*, and each node has a constant potential energy *ε*. Now we introduce two types of single-point defects in this model, one being a position defect that has a different potential energy *α* at node *j*_*d*_ and the other as a transition defect, where a distinctive transition rate *β* is assigned. Without loss of generality, we assume that the parameters *ε*, *γ*, *α* and *β* are reals. To account for these defects, the system Hamiltonian is modified as


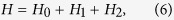


with


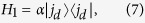



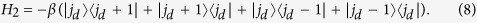


The position energy at the defect node *j*_*d*_ is *ε* + *α* and the transition rate between it and its neighboring nodes is *γ* + *β*.

### Eigen problem of the model Hamiltonian

The Hamiltonian of the CTQW on an uniform infinite line is invariant under spatial translation. Consider the discrete translational operator **T**_*n*_, which acts on the node states such that 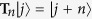. This operator is unitary, and as such can be written in the form **T**_*n*_ = *e*^*i***k***n*^, where **k** is an Hermitian operator and the generator of the translation. In the case where the Hamiltonian is invariant under spatial translation, the Hermiticity of **k** indicates that its eigenstates 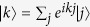 form a complete orthonormal basis, satisfying the eigenvalue equation 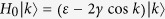, where 0 ≤ *k* ≤ *π*. The addition of a defect breaks the translational symmetry of the system, which results in an emergence of localized eigenstates of the corresponding quantum walk. The eigenstates of CTQW on a infinite line with a single-point defect can be obtained by solving a set of recurrence equations as the following.

Expanding the eigenstate 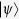 of *H* in the position space as 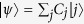 and substituting it into the eigen equation 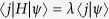 with eigenvalue *λ*, we get a set of recurrence equations about *C*_*j*_


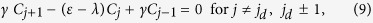



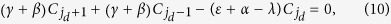



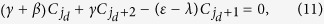



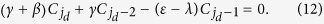


The general solution of Eq. (9) is


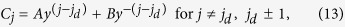


where *A* and *B* are arbitrary constants, and *y* satisfies the following equation


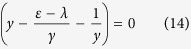


Solving the above equation yields 
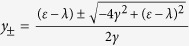
. It can be easily shown that 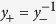, and therefore we only need to substitute *y* = *y*_+_ into Eq. (13) as our general solution.

Due to the reflection symmetry of the underlying potential with defects at a single node *j* = *j*_*d*_, the system eigenvectors in position space must possess either an odd or even parity at the defect node. In the case of odd parity, i.e. 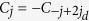, we let 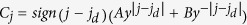, Substituting this into Eqs. (9, 10, 11, 12) and using Eq. (14), we obtain the coefficients as


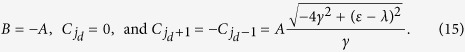


In the case of even parity, i.e. 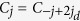, we let 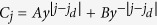 and the coefficients are


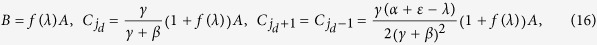


where


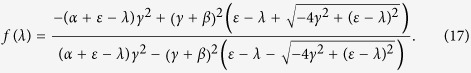


The arbitrary constant A in Eqs. (15) and (16) will be determined by the normalized condition of the state vector.

The eigen vectors are traveling waves or bound states in position space are modulated by the module value of y, which depends on the eigenvalues of the system. When *λ* ∈ [*ε* − 2|*γ*|, *ε* + 2|*γ*|], |*y*| = 1 and we can set *y* = *e*^*ik*^. The solution given by Eq. (13) is thus a traveling wave, and the corresponding eigenvalue *λ* can be obtained from Eq. (14)


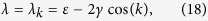


where *k* is analogous to the wave number of free particle in period lattice. Substituting Eq. (18) into Eq. (15) and Eq. (16), we get the normalized odd-parity traveling eigenvector


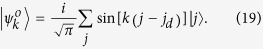


and even-parity traveling eigenvector


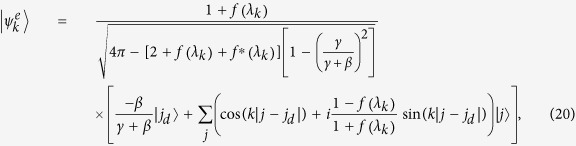


respectively, where


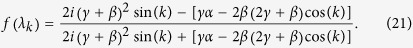


We note that odd-parity traveling eigenvector is independent on the defect parameters *α* and *β*, just like on the uniform infinite lattice line traveling with constant amplitude. It is very different for the even-parity traveling eigenvector, in which the wave traveling towards right and the wave traveling towards left have different amplitudes and they are inversion symmetry about the defect position. The amplitudes are adjusted not only by the defect parameters but also by the wave number *k*. If only *β* = 0, the the even-parity traveling eigenvector reduces to 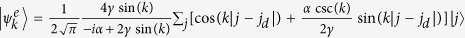 as given by Izaac *et al.*^30^. If both *β* = 0 and *α* = 0, it comes back to the free case 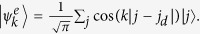


When *λ* < *ε* − 2|*γ*|, we have |*y*| > 1, and when *λ* > *ε* + 2|*γ*|, |*y*| < 1. For Eq. (13) being convergent at the infinity, either *A* or *B* must be zero. In the case of odd-parity, there is no physical solution for *C*_*j*_ due to the requirement *B* = −*A*. However, for the case of even parity, if 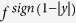
(*λ*) = 0, Eq. (13) can be reduced to 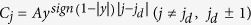, the bound eigenvector exists, and the corresponding bound eigenvalues *λ*_*b*_ can be obtained from solving the equation 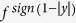
(*λ*) = 0 as


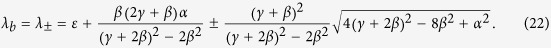


In this case the system has zero, one, or two bound eigenstates, dependent on the value range of the parameters *ε*, *γ*, *α* and *β* to satisfy with |*y*| > 1 or |*y*| < 1. Other coefficients in Eq. (16) are found to be 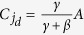 and 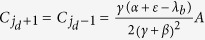. Finally, the normalized bound eigenvector with even parity can be written as


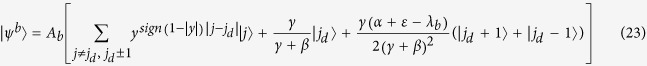


with


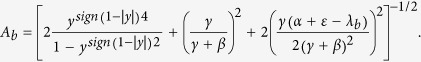


Its distribution on the position space is centered at the defect node, and exponentially decays with increasing of the distance from defect node. The height of the center peak and the decaying rate are determined by the strength of the defect.

Using the orthogonality relations of the sine and cosine functions, it can be easily shown that 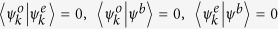 for all values of 0 ≤ *k* ≤ *π*, and 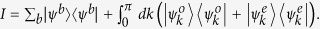 That is to say, the eigenvectors obtained above remain orthonormal with respect to each other and they form a complete set of basis. Consequently, the time-evolution of an arbitrary initial state 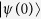 can be constructed in an integral form as


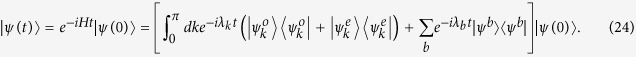


We have verified numerically in the following calculation that the integral result given by the above equation is completely consistent with that obtained by taking the matrix exponential of the Hamiltonian directly from Eq. (4).

### The effect of a position defect

**Figure 1:**
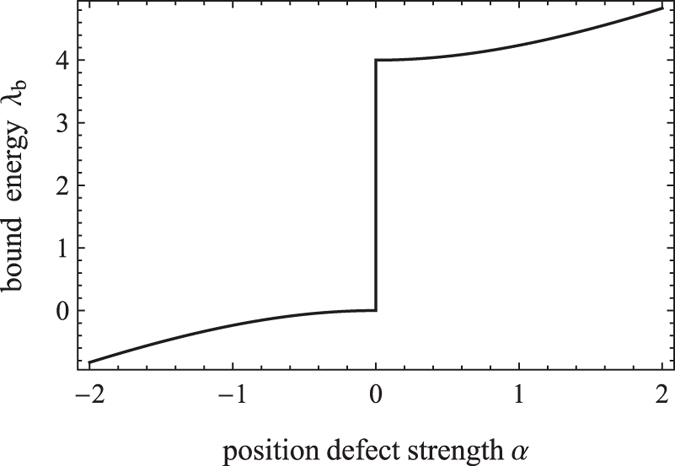
The variation of bound energy with the strength of position defect.

Choosing the parameter values *ε* = 2, *γ* = 1 and *β* = 0, we firstly examine the effects of a position defect on the quantum walk. In this case, there is always one bound state as long as *α* ≠ 0. The bound eigen energy *λ*_*b*_ as a function of *α* is shown in Fig. 1, in which *λ*_*b*_ = *λ*_+_ > *ε* + 2*γ* if *α* > 0 or *λ*_*b*_ = *λ*_−_ < *ε* − 2*γ* if *α* < 0.

**Figure 2:**
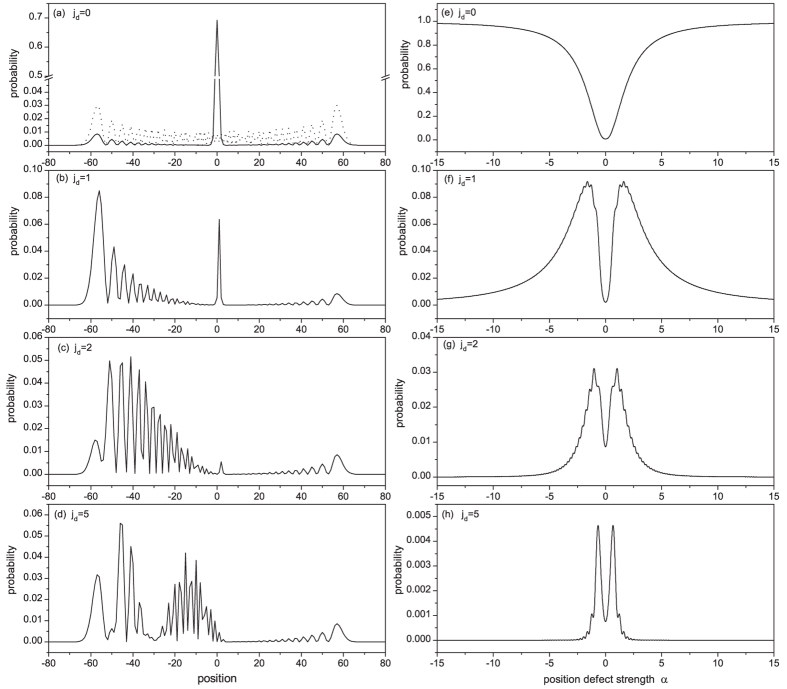
Left panel: the probability distribution of CTQW with a single-point position defect when *t* = 30, *α* = 3, *j*_0_ = 0 and *j*_*d*_ = 0, 1, 2, 5; Right panel: the probability at the defect position as a function of position defect strength.

The left panel of Fig. 2 shows the CTQW probability distribution at *t* = 30, given that the quantum walk initially starts at the origin *j*_0_ = 0, the strength of defect *α* = 3, and the defect position *j*_*d*_ = 0, 1, 2, 5, respectively. If a defect is located at the initial position *j*_*d*_ = *j*_0_, a large sharp peak appears at this position (see Fig. 2(a)) and its height remains largely unchanged with time. For comparison, the dashed line depicts the probability distribution of the free quantum walk without the defect. When the defect position is the nearest to the initial position of CTQW, i.e. 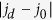 = 1, the probability distribution also has a small peak localized at the defect position (see Fig. 2(b)). However, when the defect position deviates away the initial position more a little, i.e. 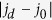 > 1, the CTQW probability at the defect position decrease rapidly to a minimum (see Fig. 2(c,d)). This phenomenon is related to the bound state induced by the presence of a single defect. It can be readily illustrated through decomposed form of the CTQW probability at the defect position


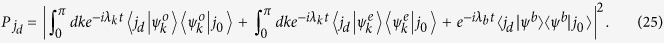


The first term in the sign of absolute value is zero forever due to 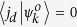 in Eq. (19). With the changes of *j*_*d*_ − *j*_0_, the probability deriving from the second term has larger amplitudes at the tails of its distribution, just similar to the probability distribution of the free quantum walk induced by the interference of traveling waves. Unlike that, the probability deriving from the third term is mainly localized around *j*_*d*_ − *j*_0_ = 0. Compared with the third term, the second term can be neglected when the distance between the initial position and the defect position is not too large. So Eq. (25) can be approximated as


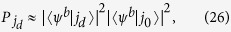


which is the combined projections of the initial position state 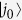 and defect position state 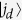 onto the bound eigenstate 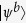. This approximation may be weakly depend on the defect parameter values and evolution time, but under our choosing parameter values they are at least different from two orders of magnitude. The height of the large sharp peak in Fig. 2(a), calculating from Eq. (25), is 0.692427, and the height of the smaller peak in Fig. 2(b) is 0.0637546, which almost agree with the approximate results from Eq. (26) 
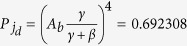
 and 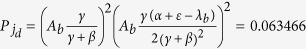, respectively. Therefore, the spike in the probability distribution at the defect position can be regarded as a fingerprint of this bound state, which can be termed as eigen-localization. When 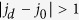, 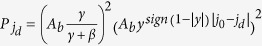 in Eq. (26) decrease exponentially with the increase of distance 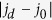 and the approximation becomes invalid. From Fig. 2(b–d), it is also observed that the CTQW is largely reflected by the defect with a small probability of transmission. Prior to encountering the defect, the CTQW is free and evolves symmetrically in both the left and right direction. Once the part moving in the right direction meets the defect, it will be largely reflected and move towards the left. As a result, two envelopes appear on the left side of the defect position and they overlap each other resulting in a complex interference pattern, as shown in Fig. 2(c,d).

The right panel of Fig. 2 shows the CTQW probability distribution at the defect position *j*_*d*_ = 0, 1, 2, 5, respectively, as a function of the defect strength *α* at *t* = 30. It is shown that, although the bound energy is less than the traveling-wave energy when *α* < 0 and greater when *α* > 0, the probability at the defect position is symmetric about *α* = 0. That is to say, CTQW treats the single-point position defect exactly the same regardless of it being a potential barrier or a potential well. When the CTQW starts from the defect position, the probability amplitude at the defect position increases monotonically with the strength of the defect potential (see Fig. 2(e)). The stronger the defect potential, the larger the probability amplitude, with the CTQW largely localized at the defect position. When the CTQW does not start from the defect position, the probability at the defect position is not monotonic but rather increases firstly and then decreases with increasing defect strength *α*. It tends to zero for the stronger defect strength.

**Figure 3:**
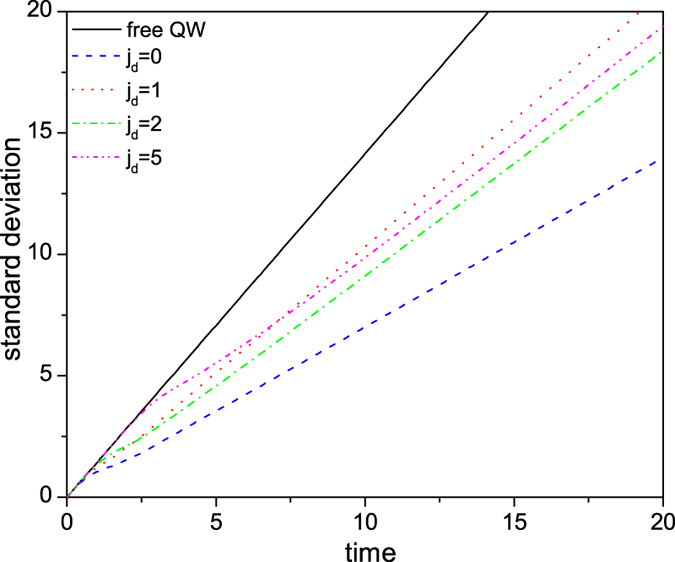
The standard deviation of CTQW with a single-point position defect as a function of time.

In addition, Fig. 2(a) shows that, besides a large peak at the origin, two smaller peaks are also observed at the tails of probability distribution, as the same locations as the ballistic peaks of the free quantum walk. Even when the CTQW starts from the left of the defect and it is largely reflected, as shown in Fig. 2(b–d), the probability distribution still has a smaller peak on the right tail. For illustrating how a single-point position defect affect the CTQW spreads on the line, we plot the variation of CTQW’s standard deviation 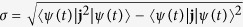 with time *t* in Fig. 3, which demonstrates predominantly a linear relationship regardless of being localized or reflected by the defect. However, the spreading speed (given by the slope of standard deviation with time) is dependent on the position of the defect. The appearance of defect makes the standard deviation less than that of a defect free CTQW. As expected, for the case *j*_*d*_ = *j*_0_ the spreading speed is the least due to strong localization. The pink dash-dot-dot line of 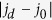 = 5 clearly shows that the CTQW spreads like a free QW at the beginning, but when it encounters the defect the spreading speed starts to decrease. In general, the larger the distance 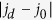, the greater the spreading speed. As an exception, we observe a much higher spreading speed for the case 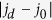 = 1 (the red dotted line in Fig. 3) due to the large reflected peak at the far left end, indicating strong interference and resonance for this special case.

### The effect of transition defect

**Figure 4:**
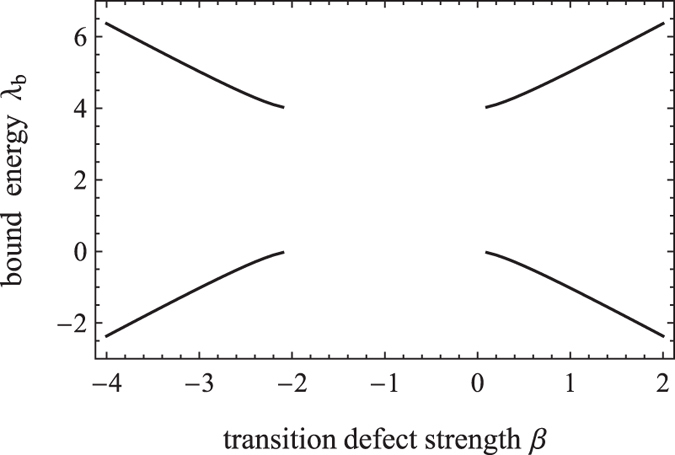
The variation of bound energy with the strength of the transition defect.

In this section, we focus on the effect of a single-point transition defect on the spreading properties of CTQW. We choose the parameters *ε* = 2,*γ* = 1 and *α* = 0, the bound energy as a function of transition defect strength is shown in Fig. 4. When |*γ* + *β*| ≤ 1 (i.e. −2 ≤ *β* ≤ 0), no bound eigenstate exists, or else there are two bound states.

**Figure 5:**
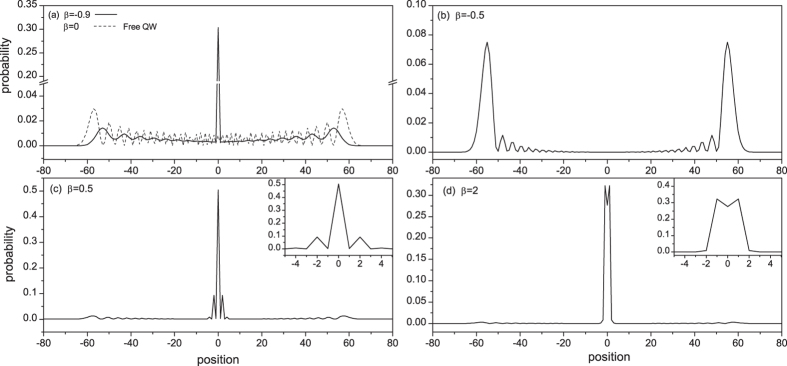
The probability distribution of CTQW with a single-point transition defect when *t* = 30, *j*_*d*_ = *j*_0_ = 0, and *β* = −0.9, −0.5, 0.5, 2.

When the defect is located at the initial position (*j*_*d*_ = *j*_0_ = 0), the resulting probability distribution over the discrete position space at time *t* = 30 is shown in Fig. 5. Some important features to note: (1) if (*γ* + *β*) = 0, the initial position is disconnected from its neighbors and consequently the CTQW stays at the initial position; (2) as |*γ* + *β*| deviates slightly from zero, the residual effect of the disconnection still shows and the probability distribution has a peak at the initial position (see Fig. 5(a)); this peak decreases with time, which distinguishes it from the localized peak induced by eigen bound state; (3) as |*γ* + *β*| increases until it approaches 1, the CTQW spreads in a similar way as a free QW since there is no bound state yet (see Fig. 5(b)); and (4) when |*γ* + *β*| > 1 (e.g. *β* = 0.5 and 2, as shown in Fig. 5(c,d) respectively), the transition defect induces two bound states surrounding the defect, resulting in a large probability in the vicinity of the defect position due to eigen-localization.

Unlike the position defect induced localization where the maximum of probability is always at the defect position, the maximum probability induced by a transition defect may also be at the defect neighbors (see the insert in Fig. 5(c,d)), which is resulted by interference between the two bound states. Neglecting the contribution from traveling eigen state, the localization probability around defect position (*j*_*d*_ = *j*_0_) can be approximately expressed by


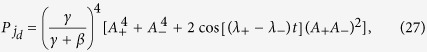



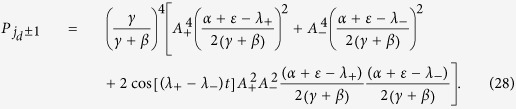


**Figure 6:**
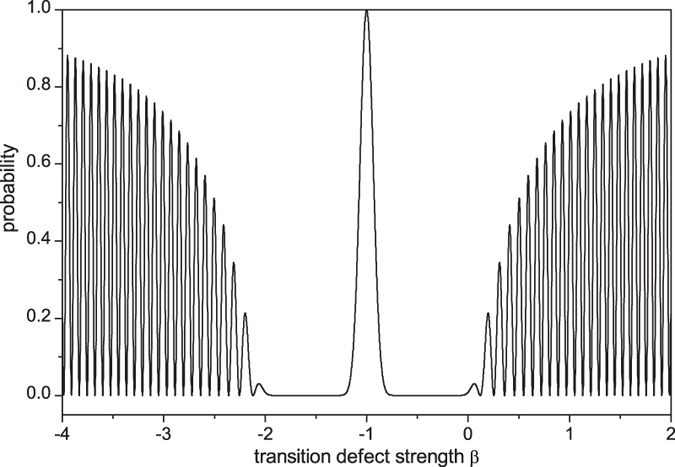
The probability at defect position as a function of the transition defect strength when *t* = 30 and *j*_*d*_ = *j*_0_.

The last terms in the square brackets of the above equations represent the interference between the two bound states. The values of Eqs. (27) and (28) are approximately equal to the peak values in Fig. 5(c,d), fully indicating that these peaks are the eigen localization. In Fig. 6, we plot the localized probability at defect position as a function of the transition defect strength *β* when *j*_*d*_ = *j*_0_. The oscillatory behavior in the range of |*γ* + *β*| > 1 displays clearly the coherent effect between the two bound states. Similar oscillation also occurs for the probabilities at the neighbors of the defect position. When *β* = −*γ* = −1, complete disconnection between the initial position and its neighbors, we have 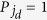. Smooth variation of 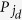 with the small deviation from *β* = −1 indicates the disconnection effect remains.

**Figure 7:**
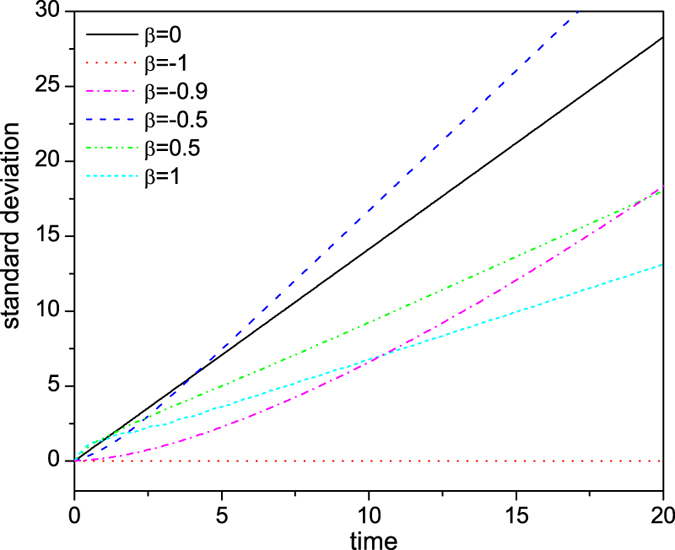
The standard deviation of CTQW with a single-point transition defect as a function of time.

The influence of a transition defect on the spread speed of CTQW is shown in Fig. 7 through the variation of its standard deviation with time. One particular interesting case is *β* = −0.5, where the spreading speed is significantly larger than that of a defect free CTQW, due to constructive interference caused by the defect. In general, however, the transition defect reduces the spreading speed due to eigen-localization and transition defect trapping. Also, when |*γ* + *β*| deviates slightly from zero (e.g. *β* = −0.9), the variation of standard deviation is clearly non-linear. This is because the residual disconnection effect decreases with time, as the probability remaining at the initial position decreases, and correspondingly the spreading speed increases.

**Figure 8:**
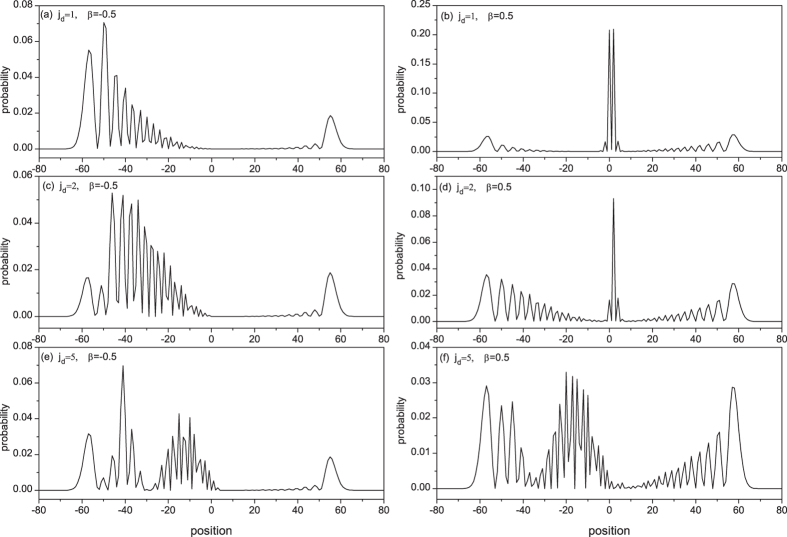
The probability distribution of CTQW with a single-point transition defect when *t* = 30, *j*_0_ = 0, *j*_*d*_ = 1, 2, 5 and *β* = −0.5, 0.5.

When the CTQW does not start from the defect position, i.e., *j*_*d*_ ≠ *j*_0_ = 0, Fig. 8 presents the probability distribution at time *t* = 30. The left panel, with *β* = −0.5 and thus no bound state existing, shows that the CTQW wave-packet is largely reflected with a smaller transmission peak observed at the same locations as the ballistic peaks of the free quantum walk. The right panel is the situation for *β* = 0.5, where two bound state exist. If the defect position is the nearest to the initial position of the CTQW, *j*_*d*_ = *j*_0_ + 1, the eigen localization induced by two bound states accumulates the probability in the vicinity of the defect position and displays strong eigen-localisation (see Fig. 8(b)). Only considering the projections of the bound eigenvectors, we have 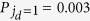 and 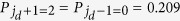, which is nearly equal to the coordinate values in Fig. 8(b). If the defect position goes away from the initial position, 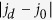 > 1, the factor 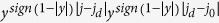 in combinated projection 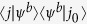 makes the eigen-localization probability decay exponentially with increasing distance 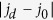.

## Discussion

We have introduced a new form of defects in continuous time quantum walks, namely a single-point transition defect. A complete set of analytical eigenvectors in position space for CTQW on the line with a single -point position defect and a single-point transition defect is obtained. While the system containing only a single-point position defect has one bound state, the system possessing a single-point transition defect has zero, one, or two bound states dependent on the transition defect parameters. With these bound eigenstate solutions we are able to understand the detailed dynamical properties of CTQW, including transmission, reflection and localization. We found that the induced localization at the defect position is determined by the combined projections of the initial position state 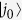 and defect position state 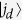 onto the bound eigenstate 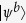. Also, the coherent effect between two bound eigenstates can be identified through the oscillating eigen localization for the case of single-point transition defect. We present a particularly interesting case where, due to constructive interference caused by the defect, the spreading speed is significantly larger than that of a defect free CTQW. This study provides another way of controlling the scattering properties of quantum walks by introducing transition defects besides the previously studied position defects.

This kind of eigenstate localization is different from the Anderson localization of CTQWs. The Hamiltonian in the Anderson model are randomly chosen whereas the Hamiltonian under our consideration is deterministic. The propagation behavior for a system which exhibits Anderson localization is that for any initial state and an arbitrary number of time steps, and the probability to find the particle at a position is upper bounded by an almost exponentially decaying function in the distance from its initial position. The eigenstate localization for our model considered depend strongly on the initial state of the quantum walker, more precisely on the distance between the defect position and the initial position. In fact, there are initial states such that the propagation behavior is ballistic in the sense that the variance of the particle’s position distribution grows quadratically with time. The single-point defects in our model, as a local modification, can be regarded as a perturbation of a translationally invariant Hamiltonian and such perturbations generically generate bound eigenvectors. The peak in the probability distribution, occurring around the defect, can be understood as eigen-localization, which should be also allowed for high dimensions.

## Additional Information

**How to cite this article**: Li, Z. J. and Wang, J. B. Single-point position and transition defects in continuous time quantum walks. *Sci. Rep.* **5**, 13585; doi: 10.1038/srep13585 (2015).

## References

[b1] Aharonov Y., Davidovich L. & Zagury N. Quantum random walks. Phys. Rev. A 48, 1687–1690 (1993). 9909772 10.1103/physreva.48.1687

[b2] Farhi E. & Gutmann S. Quantum computation and decision trees. Phys. Rev. A 58, 915–928 (1998).

[b3] Kempe J. Quantum random walks - an introductory overview. Contemp. Phys. 44, 307–327 (2003).

[b4] Oliveira A. C., Portugal R. & Donangelo R. Decoherence in two-dimensional quantum walks. Phys. Rev. A 74, 012312 (2006).

[b5] Kurzyński P. & Wójcik A. Discrete-time quantum walk approach to state transfer. Phys. Rev. A 83, 062315 (2011).

[b6] Crespi A. *et al.* Anderson localization of entangled photons in an integrated quantum walk. Nat. Photonics 7, 322–328 (2013).

[b7] Kitagawa T. *et al.* Observation of topologically protected bound states in photonic quantum walks. Nat. Commun. 3, 882–889 (2012). 22673909 10.1038/ncomms1872

[b8] Bhattacharya N. *et al.* Implementation of Quantum Search Algorithm using Classical Fourier Optics. Phys. Rev. Lett. 88, 137901 (2002). 11955125 10.1103/PhysRevLett.88.137901

[b9] Childs A. M. Universal Computation by Quantum Walk. Phys. Rev. Lett. 102, 180501 (2009). 19518851 10.1103/PhysRevLett.102.180501

[b10] Du J. *et al.* Experimental implementation of the quantum random-walk algorithm. Phys. Rev. A 67, 042316 (2003).

[b11] Schmitz H. *et al.* Quantum Walk of a Trapped Ion in Phase Space. Phys. Rev. Lett. 103, 090504 (2009). 19792773 10.1103/PhysRevLett.103.090504

[b12] Karski M. *et al.* Quantum Walk in Position Space with Single Optically Trapped Atoms. Science 325, 174–177 (2009). 19589996 10.1126/science.1174436

[b13] Broome M. A. *et al.* Discrete Single-Photon Quantum Walks with Tunable Decoherence. Phys. Rev. Lett. 104, 153602 (2010). 20481989 10.1103/PhysRevLett.104.153602

[b14] Schreiber A. *et al.* Photons Walking the Line: A Quantum Walk with Adjustable Coin Operations. Phys. Rev. Lett. 104, 050502 (2010). 20366754 10.1103/PhysRevLett.104.050502

[b15] Sansoni L. *et al.* Two-Particle Bosonic-Fermionic Quantum Walk via Integrated Photonics. Phys. Rev. Lett. 108, 010502 (2012). 22304249 10.1103/PhysRevLett.108.010502

[b16] Schreiber A. *et al.* Decoherence and Disorder in Quantum Walks: From Ballistic Spread to Localization. Phys. Rev. Lett. 106, 180403 (2011). 21635071 10.1103/PhysRevLett.106.180403

[b17] Kendon V. & Tregenna B. Decoherence can be useful in quantum walks. Phys. Rev. A 67, 042315 (2003).

[b18] Annabestani M., Akhtarshenas S. J. & Abolhassani M. R. Decoherence in a one-dimensional quantum walk. Phys. Rev. A 81, 032321 (2010).

[b19] Yin Y., Katsanos D. E. & Evangelou S. N. Quantum walks on a random environment. Phys. Rev. A 77, 022302 (2008).

[b20] Mülken O. & Blumen A. Continuous-Time Quantum Walks: Models for Coherent Transport on Complex Networks. Phys. Rep. 502, 37–87 (2011).

[b21] Ribeiro P., Milman M. & Mosseri R. Aperiodic Quantum Random Walks. Phys. Rev. Lett. 93, 190503 (2004). 15600819 10.1103/PhysRevLett.93.190503

[b22] Keating J. P. *et al.* Localization and its consequences for quantum walk algorithms and quantum communication. Phys. Rev. A 76, 012315 (2007).

[b23] Kollár B. *et al.* Asymptotic Dynamics of Coined Quantum Walks on Percolation Graphs. Phys. Rev. Lett. 108, 230505 (2012). 23003932 10.1103/PhysRevLett.108.230505

[b24] Chandrashekar C. M. Disordered-quantum-walk-induced localization of a Bose-Einstein condensate. Phys. Rev. A 83, 022320 (2011).

[b25] Wójcik A. *et al.* Trapping a particle of a quantum walk on the line. Phys. Rev. A 85, 012329 (2012).

[b26] Li Z. J., Izaac J. A. & Wang J. B. Position-defect-induced reflection, trapping, transmission, and resonance in quantum walks. Phys. Rev. A 87, 012314 (2013).

[b27] Zhang R., Xue P. & Twamley J. One-dimensional quantum walks with single-point phase defects. Phys. Rev. A 89, 042317 (2014).

[b28] Motes K. R., Gilchrist A. & Rohde1 P. P. Quantum random walks on congested lattices. *arXiv:1310.8161*. 10.1038/srep19864 PMC4728491 26812924

[b29] Koster G. F. & Slater J. C. Wave Functions for Impurity Levels. Phys. Rev. 95, 1167 (1954).

[b30] Izaac J. A., Wang J. B. & Li Z. J. Continuous-time quantum walks with defects and disorder. Phys. Rev. A 88, 042334 (2013).

[b31] Manouchehri K. & Wang J. B. Continuous-time quantum random walks require discrete space. J. Phys. A 40, 13773–13785 (2007).

